# How to make an app-based program work and show how it works

**DOI:** 10.1371/journal.pgph.0005034

**Published:** 2025-09-10

**Authors:** Ricky Janssen, Nora Engel, Anja Krumeich, Aliasgar Esmail, Keertan Dheda, Réjean Thomas, Nitika Pant Pai

**Affiliations:** 1 Department of Health, Ethics and Society, Care and Public Health Research Institute (CAPHRI), Maastricht University, Maastricht, The Netherlands; 2 Mental Health and Neuroscience Research Institute (MHeNs), Faculty of Health, Medicine and Life Sciences, Maastricht University, Maastricht, The Netherlands; 3 Athena Institute, Faculty of Science, Vrije Universiteit Amsterdam, Amsterdam, The Netherlands; 4 Division of Pulmonology, Department of Medicine, Centre for Lung Infection and Immunity, University of Cape Town, Cape Town, South Africa; 5 Faculty of Infectious and Tropical Diseases, Department of Immunology and Infection, London School of Hygiene and Tropical Medicine, London, United Kingdom; 6 Clinique Médicale L’Actuel, Montréal, Canada; 7 Division of Clinical Epidemiology, Department of Medicine, McGill University and Research Institute of the McGill University Health Centre, Montréal, Canada; Tribhuvan University Institute of Medicine, NEPAL

## Abstract

For digital health interventions, the “gold standard” of evaluating effectiveness is the randomized control trial (RCT). Yet, RCT methodology presents issues such as precluding changes to the technology during the study period as well as the use of study settings that do not reflect “real world” contexts. In this paper, we draw on empirical material from our ethnographic research on an app-based program called HIVSmart!, which is a digital strategy designed to support people in the process of HIV self-testing. We explore how this digital health approach was made to work in different contexts, and what this means for monitoring and evaluation of digital health interventions. Our analysis reveals that making technology which is “ease to use” depends on various user practices developed over time and the multiple, shifting users who define this ease of use. Furthermore, it shows that asking whether an app is “working” provides different answers at different moments, as the technology, as well as the human and physical supports around it, change and adapt over time. Therefore, questions around the feasibility of the technology, as well as ongoing monitoring efforts, need to be developed in a way that capture this fluctuation in the technology and its surroundings. Based on our insights, we contribute to implementation science approaches in the field of digital health by putting forward recommendations regarding how we can assess the effectiveness of digital health tools.

## Introduction

Health interventions increasingly integrate innovative digital components such as apps, websites, and sensors (in wearables). When implementing health care innovations and moving them between contexts, it is important to establish whether the innovation is working and effective. Specific monitoring and evaluation efforts aim at assessing the impact of new digital health interventions and whether they work as intended by their designers. Guidelines from the World Health Organization specify how to conduct such evaluations;

“The evaluation of the digital health system and intervention over time is an attempt to attribute a range of outcomes to the technology-based intervention – from assessing how easily end-users can interact with the system (usability), to the health impacts attributed to the intervention (efficacy/effectiveness), to the affordability of the system (economic/financial evaluation). In later stages of maturity, questions may arise around the integration of the system and its data streams within the broader health system architecture and policy environment, as interventions attempt to reach and sustain national scale (implementation science).” [1] (p2)

These guidelines provide input for researchers looking to design and carry out monitoring and evaluation activities for digital health interventions. They specify the role of quantitative and qualitative methods and point to several stages related to the maturity of a digital health intervention. These stages include assessing:

1. Feasibility (does the digital health system work as intended in a given context)
2. Usability (is the digital health system used/interacting as intended)
3. Efficacy (does it achieve the intended results in a controlled setting)
4. Effectiveness (does it achieve intended results in an uncontrolled setting) and;
5. Implementation research (uptake, institutionalization, and sustainability of the intervention in a given setting)

For the case of digital health evaluation, the “gold standard” of evaluation from the stage of feasibility through effectiveness is the randomized control trial (RCT) [1]. But RCT methodology presents issues such as precluding changes to the technology during the study period, lack of variation in study sites that do not reflect real-world contexts, trial duration that is too long to keep pace with rapid technology development [2] but too short to account for long-term effectiveness of interventions, as well as potential ethical and feasibility issues [1]. During the implementation stage, the WHO guidelines indicate multi-centre quasi-experimental design as the method which provides the strongest evidence about an intervention. As we will illustrate in this paper, doing monitoring and evaluation across the phases of maturity of digital health technology requires work, flexibility and creativity from technology developers, researchers and health professionals in order to respond to the challenges of situatedness. The word ‘situated’ refers to the local or context specific way something happens, such as situated knowledge creation or situated actions [3,4]. This multiplicity or situatedness is not confined to the implementation stage alone. However, in current global health metrics, multiplicity is reduced to universal, standardized and uniform ways of counting and understanding interventions and their effectiveness [5]. Social scientists in the field of Global Health have called on those who carry out clinical trials and implement global health interventions to acknowledge and reflect on the situatedness of interventions, including how they work in different contexts, and how local characteristics contribute to their effectiveness [6,7]. “The challenge for global health trials is to acknowledge the value in local specificity and rather than seeking to efface it, work with it to produce science that is both rigorous and situated.” [7] (p39).

Greenhalgh and Russell [8] critique the use of scientific approaches, whose focus on variables with clear boundaries and causative effects, remove the contextual elements that help explain the success or failure of an intervention. They present an “alternative set of principles” for guiding eHealth evaluation. They invite other researchers to test these principles, which emphasize, for example, emergent evaluation approaches, changing contexts, consideration of various individuals who work with a technology and what the technology means to them, as well as expectations around a technology [8]. Furthermore, Engel [9] uses the case of point-of-care (POC) diagnostics for tuberculosis and HIV to show how technology development and use are continuously engaged in a process of alignment, as the technology is reshaped to meet the visions and needs of different users. For example, a technology is designed to align to users in a particular setting, but also involves alignment with the developer of the technology, global intermediaries who set out standards, and the disease which the technology is designed to address (f.i. HIV or tuberculosis) [9].

In this paper, we draw on qualitative data from our study on HIVSmart!, an app-based self-testing platform (see Box 1). It was developed and evolved over time into a program – with an app, a web-program, a dashboard, a notification service and more (see Box 1). Here we aim to address the following two research questions: 1) how was this app-based program made to work across contexts and 2) how is this (not) captured in monitoring and evaluation of the digital health intervention? In order to answer these questions, we draw on Engel’s conceptualization of technology development and implementation to investigate the shifting users and ongoing process of alignment involved in the development and use of HIVSmart! for HIV self-testing. In addition, following the principles of Greenhalgh and Russell, we consider the expectations and limitations inscribed into HIVSmart!, the individuals who engage with the app-based program and how they understood the role of the technology, and how they imagined the future use of the technology. Furthermore, we identify different moments in the study which illustrate the messiness and unpredictability of the technology in practice [8].

**Box 1 HIVSmart! Case Description**

In 2009/2010 development of a smartphone app began for HIV self-testing (HIVST). The idea for this smartphone app was conceived of by Dr Pant Pai (hereby referred to as Principal Investigator) in 2009 and was further developed in 2010, together with her team of designers, researchers and patient advocates. This app and associated web program is called HIVSmart!. HIVSmart! was designed to assuage fears from the medical community regarding people testing for HIV on their own. It was designed to walk testers through certain steps of doing an HIV self-test and to provide HIV information, counselling, risk-staging and linkage to care.

After initial usability and feasibility studies in Montreal [10], the app evolved into an app and web program which was then piloted in a population of health care workers in Cape Town for feasibility [11]. The smartphone app and program was protected in 2013 and was the first in the self-testing space to offer a complete program for self-testing. It provided detailed steps on performing an HIV self-test together with evidence-based HIV information, allowed participants to stage their own risk for HIV (prior to testing) and also helped them link to counselling and care post self-test. At that time, there were no guidelines in place for these kinds of digital approaches and there was little acceptance around digital self-testing models. The app and digital web program won an open access global innovation award in 2013, which allowed the program to be taken for field-based evaluations in two countries: in Canada, in a community clinic which served the gay and MSM community [12] and in South Africa, in at-risk populations attending township-based community clinics [13]. As the app was taken from one country context to another (Canada to South Africa), and it evolved from an app into an app-based program, the Principal Investigator realized the need for additions – it had to meet the contextual needs of the country, different cultures, the technical and health systems, end users, and various other stakeholders. The program evolved from an app to include additions such as a dashboard, backend support, a front-facing text-based services program and a user interface including a physician avatar and translation of information into five languages. These additions were key to its uptake and success. Its successful deployment involved coordination between teams of people across disciplines including clinicians, technical developers, quantitative evaluators, and qualitative researchers.

## Methods

We analyzed data collected as part of an ethnographic study conducted in 2017 and 2018 on a quasi-randomized trial implementing an app-based program named HIVSmart! in Cape Town, South Africa. An RCT, by virtue of randomization, does not allow for choice and flexibility of use, or real-world observation (due to the need for concealment of intervention allocation). Hence, the PI and her team chose a quasi-randomized study design for the study in Cape Town, in order to provide flexibility to patient participants and to allow for observation and use of the app in a real-world setting. We conducted this qualitative study in parallel with the quantitative study to explore the objectives outlined above. We also analyzed empirical material gathered from interviews with study staff in Montreal in 2018, where a previous study on HIVSmart! had been conducted. Further details regarding the quasi-randomized trial in Cape Town [13] and the feasibility study in Montreal [12] are published elsewhere. In total, the study included 59 interviews with study staff and participants between the two settings, in addition to observation, informal conversations and participation in team meetings within the quasi-randomized study in Cape Town. Our previously published articles, which are based on this data, focused heavily on the experience and practice of using the app-based program and doing HIVST from the perspective of study participants [14–16]. In this article, we instead focused on the observations, fieldwork notes, interviews and focus group discussion with study staff in South Africa, as well as interviews with study staff in Montreal. This analysis draws on qualitative data including nine interviews and one focus group with relevant stakeholders such as nurses, clinic staff, health care workers and medical officers who had previously worked on, or were currently working on, the HIVSmart! study. The interviewer employed several strategies during data collection to help ensure data and interpretation quality. During interviews and discussions with healthcare staff, the interviewer, where necessary, asked clarification questions and intermittently summarized or repeated responses back to participants to check for accuracy. In addition, due to the ethnographic nature of this research, the interviewer could relate responses, topics and themes from interviews to what was seen in real-time observation during the study. Furthermore, the interviewer discussed and reflected on preliminary findings with local study staff and co-authors, who were also healthcare providers, to help ensure appropriate interpretation of data.

We employed a thematic content analysis approach to analyze the data, which includes: 1) getting to know the data through listening to interviews, rereading interview transcripts and reviewing fieldwork notes; 2) identifying themes; 3) coding the data and; 4) organizing the coded data to identify overarching themes and relationships within and across codes [17]. We used NVivo9 software to aid this coding and analysis process. Having done multiple iterations of coding between fieldwork trips, and while writing the other related articles, we now attend to some of the broader themes identified regarding the overall functioning of the HIVSmart! study. Following the approach of Greenhalgh and Russell [8], our analysis involved zooming in and out of narratives that arose through the data, while also reflecting on how these narratives challenged, and created tensions with, current methods of study design and evaluation for digital health technology.

### Ethics statement

The HIVSmart! Study in South Africa was approved by the McGill University Health Centre Research Ethics Board, Montreal, Canada and the Faculty of Health Sciences Human Research Ethics Committee at the University of Cape Town, Cape Town, South Africa. The HIVSmart! Study in Montreal was approved by the McGill University Health Centre Research Ethics Board, Montreal, Canada. Participants were recruited between July 1^st^ 2016 and February 1^st^ 2017 for the Montreal project and between January 1^st^ 2017 and June 1^st^ 2018 for the South Africa project. Written informed consent was obtained from study participants.

## Results

The results section is divided into three main themes. The first theme focuses on the feasibility of the digital health intervention and how this feasibility is shaped by changes to the app-based program and its surroundings; the second focuses on the app-based program’s usability as it relates to ease of use, provision of health information and the healthcare team it works within; and the third focuses on how the app-based program was made efficacious/effective in a particular setting.

1.1 Feasibility and the Changing App

Feasibility relates to whether a given digital health intervention works in a given context. HIVSmart! goes through different iterations over time to respond to the needs of people using the app in a particular setting (see Box 1). For example, one issue which arises is that the application is too big (i.e., it takes up too much space on people’s phone in terms of bandwidth). This is highlighted by one of the study’s medical officers in early 2017:

“[...] I was told that in some cases, the Facebook app had to be sort of deleted from the patient’s phone and then, because the available RAM was not enough to run the app. Or you download the app but it could not run properly. So things like that. I think if you can make the app smaller it might, might work better.” (Medical Officer 1)

To address this issue and make the intervention feasible, the app designer and developer worked to shrink the app. For the new smaller app, there are separate versions for each language including, for example, English, Xhosa and Afrikaans, to save space, and an additional smaller “universal” language version. This requires that the nurse or healthcare worker (HCW) choose a specific language version (e.g., English or Xhosa) or the universal version to download on the participants’ phone. However, during the focus group discussion with nurses and HCWs, the participants mentioned that this also caused further unforeseen complications in practice for the study team. The new, smaller app version had several new glitches and required more effort from the study nurses/HCWs when helping clients either download or use the app. To deal with this, the study staff tried downloading different versions of the app, they learned which phone models worked or caused problems, and they tried re-downloading the app. This shows that the study team is constantly tinkering with the technology and they adapt their practice of setting up the study participants with the technology, based on the evolving app. The app is not a stable thing throughout the study: it develops in an iterative process as it changes in response to the feedback and practices of study staff, participants, the app designer and the developer making the actual changes to the app.

1.2 Feasibility and the Changing Environment

The feasibility, and thus, imagined use of the app-based program, and how it is made to work in practice, changes in relation to its surrounding environment. In the case of the HIVSmart! quasi-randomized study, this includes changes to national-level regulatory aspects of self-testing, the various smartphones (or lack of phones) brought into the study by participants, shifting technology and supports provided by the study to accommodate participants, and shifting spaces (Montreal or Cape Town, home or at the clinic) in which the app and self-test were being used.

To begin, the fact that HIVSmart! could be used at home hinged on the approval of home HIV self-test kits by the government in South Africa, which happened in 2016 – this was not possible in Canada at the time of the initial study in Montreal. Furthermore, as the study in Cape Town progressed, tablets were provided for those without a phone or for those bringing in various phones that could not download the app. Spaces in private rooms/kiosks were provided at the clinic sites to accommodate those who needed to use a tablet, for those who were afraid to test alone, and for those who wanted to test alone at home but did not have a private space. Self-testers who tested alone with their own phones were asked to use their phones in any private space to accommodate their needs. These changes enabled the app-based program to keep working.

Feasibility asks the question – *does the technology work as intended*? However, as we highlight above, the technology, and the environment it works in, are not static. Therefore, asking if the technology works provides a changing answer: the technology works, or does not work, at different moments. Designing and developing the app is an iterative process, but the process of adjusting to changes in a particular setting and aligning the surroundings to the app (f.i. people’s roles, the physical spaces and provision of additional technology) also requires an iterative approach. In the following section, we highlight the active, and continuously developing, role of healthcare staff in helping the technology fit the needs of study participants.

2.1 Usability – Making it easy

The WHO guidelines on “Monitoring and evaluating digital health interventions” [1], propose asking the following question when assessing usability: “Do the users find the technology easy to use?” (p35). However, when speaking to one of the HIVSmart! Study Coordinators at the end of the study, she explained how using the app was not just easy or difficult to use – it became easier over time as the staff became more accustomed to the app and could explain the process more clearly to participants.

“I do know it’s [using the app is] easier, before it used to take us an hour just to get people to understand it. And I think it’s also because of our understanding of the app. You know, because we also had to understand how to use the app. So I think since we know how, it’s also become easier. Because now you are able to explain to people how to use it.” (Nurse Study Coordinator 2)

This quote illustrates the amount of time and effort study staff took to ensure the participant understood how to use the app. Furthermore, it also illustrates how the process of explaining and using the app became easier, not just because the app was easy on its own, but because over time staff was able to familiarize themselves with the technology and the related procedures. How can one then say that the app alone was “easy to use”? This ease of use also hinged on 1) the knowledge and experience of the study staff and 2) the amount of time study staff invested in ensuring participants understood how to use the technology. Here we see that there are multiple app users: the study staff using the app and learning how to work with it, and then the study participants, who learn to work with the app both on their own and through their interactions with the study staff. During the FGD, while the study staff were going back and forth discussing some of the issues around the instructions in the app and how things looked to participants, one of the nurses also remarked that going through any app can be difficult the first time, but if you use it a few times you get used to things. In “real-life”, you might use an app more than once and you might use a self-test more than once. Saying an app is easy or difficult the first time it is used can also underestimate what a user does with the technology and how they learn to use or integrate a technology over time. In this way we see another (future) user – a tester who has used the app more the once.

In our own work [14] we report that the video instructions provided by HIVSmart! for doing the HIV self-test are perceived as “easy” and that the instructions in HIVSmart! were generally perceived as “simple and straightforward”. However, participants were able to watch the video (which was only available in English) in the clinic with healthcare staff before working with HIVSmart! and the self-test on their own. We also highlight the role of the study staff in simplifying the process of using the app when we discuss language preference. We point out that participants often chose the English version of the app instead of the Xhosa version because the study nurses and HCWs directed them to and because it explained things in the app more clearly. The main issue brought up by study staff and participants was that the Xhosa version of the app contained a “deep” version of the language, instead of a “light” locally spoken version of the language in Cape Town. English was often described as easier to understand, and the study staff played a role in telling participants to use the English version, as they knew through experience that it was clearer. These examples do not refute the idea that the app itself was easy to use or engage with, but it does illustrate how the support and experience of the study staff included in the app-based program played a role in making it so. The app is not a stand-alone artefact: ease of use is also linked to the people and things around it.

2.2 Usability – Making health information useful

As we show in the previous sections, it takes time and effort to align HIVSmart! to the setting and to make it “easy” for participants. Yet as we will show below, aside from being easy, usability indicators stipulate that digital health tools should also be useful in communicating health information. Examples here include the following:

“Do the users find the health information received through the digital health intervention useful?” [1](p35)

“Are the users able to communicate with the digital health system as intended? Are they responsive to the information received through the system?” [1](p35)

However, health information received by a user may also become useful because a counsellor is there to contextualize the information further. One of the study’s medical officers recognized the potential role of clinic staff in tailoring HIVSmart! messaging to the local context, while also recognizing that the app-based program shifts some of the workload away from the counsellors.

“I mean it [the app] might take away the high burden from them [the counsellors]. But I think it’s important to have the counsellor still to be able to give like specific advice. For example, let’s say an app I feel that is very generic and it’s not directed at the local community where [there] is practices of having more than one, more than one wife. So it doesn’t specifically target at that, although it does touch on it, like multiple sexual partners, but according to the local practices if you ask somebody who’s in a polygamous marriage and ask them that “do you have multiple sexual”... they will say no because they don’t view this as a... they view it as a unit. So I feel that, those kinds of things, the insights that the counsellor does bring, will actually compliment the, the messages.” (Medical Officer 1)

While one of the medical officers mentions that the app counselling is not comprehensive (i.e., it can’t do everything), both medical officers point out that, within the program, the app and counsellors complement one another. Again, this highlights how the people and technology work together to create a novel HIVST program. In the vision presented by the medical officer, the possibility that an app does not always perfectly imbibe the local context, and nuanced understandings of sexual relationships, does not necessarily become problematic or mean the app is ineffective. Rather, it means that a clinician or counsellor may be helpful in further clarifying or aligning the app’s message to the user. For example, a public health nurse working on the study in Montreal described instances where he needed to clarify risk classifications given by the app, which reflect standard HIV risk guidelines.

“[...] people really liked to have an instant um sort of feedback about what their risk are, even though sometimes they did not agree. Because they would fall automatically being high risk since they were MSM and sometimes they would disagree [with the app] because they were using always protection but they were with an HIV+ person, which his viral load was undetectable, so there’s some uh, there’s some grey area that was not covered but in most, in those cases they understood why that level [of] specificity could not be applyable [sic] to all [...].” (Study Nurse, Montreal)

When asked what he would say to participants if they disagreed with the risk classification he said:

“I would explore uh, the elements that would for them, that classify them at high risk or at low risk. So I would just like try to clarify the discrepancy and rationalize as to why the app would say that versus what they wouldn’t tell me. But other time[s] I would say well there’s a reason why you would be at high risk and just reinforce what the, what the actual algorithm would tell them.” (Study Nurse, Montreal)

One might then ask why the need for the app at all? But again, this assumes a vision of the app which is only effective if it does everything on its own in relation to the user. Yet, if we envision the app as just one actor within a broader system as part of a program [13], we might then imagine an app which encourages moments of dialogue between patients and healthcare providers that may not have occurred otherwise. It could also provide benefits such as allowing the person to think about things on their own (alone or in private) before further discussing with a healthcare provider. It may also allow the healthcare provider to home in on concerns specific to the patient. This can also work the other way around. When asked if the app had changed her role as a nurse, one of the study coordinators in Cape Town said:

“It hasn’t really changed it but it has enhanced it in a sense. If I have forgotten to say something, it will be on the app.” (Nurse Study Coordinator 2)

Study participants sometimes mentioned that the app gave them information they had not previously received during counselling for HIV testing. In a clinical context where healthcare providers have a high workload and don’t always have enough time for each patient, the app is there to add a layer of support. It is not just the app or just the healthcare provider who supports and brings about the (potentially effective) self-testing experience; it is the two working together as part of the app-based program. A “good” app may not necessarily reduce time or make counselling sessions more efficient, it might even do the opposite. However, this isn’t necessarily a bad thing. It may lead to other discussions and contribute to good care. Or, it can also lead to more time spent on other tasks (task-shifting), such as linking patients to care [13].

2.3 The App and its Colleagues

It took time for study staff to build a relationship with and trust the technology in the intervention. Study staff were initially hesitant towards the app and self-testing for different reasons. One of the fears discussed in a focus group discussion with the healthcare workers and nurses was what would happen to participants that test at home. For example, they were concerned about suicidal reactions or that participants would not return to the clinic after self-testing. One healthcare worker mentioned:

“But I’m still worried – what if we got a patient that’s gonna test positive at home. And what if, within about the app, what’s gonna happen? Are they gonna come back with the test or they’re gonna sit at home or do what themself?”

When asked what the solution was regarding the risks around doing the test at home, one nurse responded:

“I think the solution is that we um we need to really, we need to really counsel them about, we need to focus counselling, we need to counsel it about what if you can have unfavourable results, what can you do. But you have to have a support there at home. […]”

The nurse then said that the counselling in the app would re-emphasize the counselling done in the clinic. There is also ambivalence in the focus group discussion when discussing these issues – it could be scary to receive a result at home, or someone could react negatively towards a partner, but one of the nurses also mentions that a loved one may be at home to support the person.

There are also overarching concerns about whether this app-based program will take away the future jobs of the HIV counsellors who do testing at the clinic. Yet, there was ambivalence around this as well – what about those people without phones who will still do conventional testing, and what about those who need additional/post-test counselling? If the app-based program is implemented as part of the regular clinic services, people will need support to use HIVSmart!. Later in the discussion, one of the study staff also points out that the app-based program might allow counsellors more time for adherence counselling for treatment. It takes time for the staff to settle in or adjust their roles as the study moves along – their tasks shift and evolve, including things such as helping participants link to further care. Here, we see that feelings towards the technology emerge within certain (imagined) situations and that there are shifting visions around the future of care work and the app-based program.

3.1 Efficacy, effectiveness, and future expectations

Giving the study staff time to get to know the app and see how it works in practice instilled confidence in the intervention. In an interview with the study coordinator near the end of the study, when asked if she viewed the project differently now than at the beginning she said;

“Yes, yes I do because I’m more positive about it. Because I can see the results and I can see that because we’ve made sure that people are linked to care immediately. I think that people do come back – they might not come back to the same clinic, but they do come back. They do go back to another clinic.” (Nurse Study Coordinator 2)

She mentions that the app informs participants about linkage to care, however, she also highlights that this linkage to care happened largely through the additional effort of the nurses and healthcare workers in the study who contacted participants.

“Because generally when people come initially to a clinic, they do the test, and if they are positive, they kind of run away, bury their heads in the sand, and that’s it, and pretend it’s not there. Whereas with this, they actually have to come back. You know because we ask them to come back in a short while, or we call them to see how they are doing and if they’ve been linked to care. We also check to see if them um, if they have come back to the clinic. So we kind of keep a tab on them.” (Nurse Study Coordinator 2)

Pant Pai and colleagues echo this in the description of their intervention saying; “To arrange for linkages to counselling, disease staging or ART initiation in test positives and prevention services in test negatives, the healthcare workers/peer counsellors recorded participant’s language preferences, preferred mode of follow-up communication (chat, SMS, phone call and face to face) and their preferred clinic location [...]” [13](p4). The nurse mentioned they would call patients, check in with them and encourage them to come back. These connections were part of the program encompassing the app and took the work and care of the healthcare professionals surrounding it to make the linkage to care process successful.

## Discussion

Our paper shows how the challenges, successes, and envisioned uses of an app-based program designed to support HIV self-testing can become obscured through the metrics and foci used to evaluate digital health technologies and their intervention outcomes. The app-based program changes, the intervention requires modifications, and the surrounding staff and technical/physical infrastructure adjust to help the technology work within the local context. Different users interact with the app-based program and aim to do different things: the developer aims to make the app function within different technical infrastructure to provide HIVST support; the nurse or healthcare worker aims to understand how the app works to support the study participants as part of providing care; the study participants look to the app for advice and support while doing the self-test. But, how is this reflected in metrics used to monitor or evaluate these interventions? The WHO guidelines state that metrics should show changes that occurred as a result of a digital health intervention. Yet, research on barriers to POC testing shows that the availability of rapid or simple diagnostic tests alone does not define POC testing, rather it is the successful use of testing technology in a POC testing *process* that matters [18]. This means that evaluation of digital health technology should not be focused on just the technology itself, the technical system which allows it “to work” in a particular context, or the usability of the technology in relation to a singular user. Rather, we need to evaluate the program encompassing the app/digital technology, which together forms a changing clinical service delivery process (in our case, this is also diagnostic in nature). As our results on the app-based program highlight, we should not only ask, for example, “was the app easy to use” – yes or no? How or why? We should also take a much closer look at what makes the process of using the technology easy over time and the multiple, shifting users who define this ease of use. For example, we should ask:

- Who exactly finds the app easy to use?
- What other things or people facilitate the interaction between the person and technology?
- How, if at all, did these people or things contribute to ease of use?

Papoutsi and colleagues [19] show how the, sometimes invisible, work of nurses to troubleshoot problems during a co-design project for remote monitoring technology are understood as quick-fix solutions within a study, rather than important actions that should inform the co-shaping of a technology-supported service. Building on these insights, we recognize the effort of nurses and healthcare workers, as well as the additional resources, necessary to make the intervention successful in terms of health outcomes. If a technology is envisioned as part of a broader service, which is changeable in nature, we must then evaluate the technology and measure outcomes as part of the broader services in which it is embedded.

This study has implications for how we understand and measure the resources needed to make a digital health intervention work. Learning how to use a digital health tool, and the co-development of practices between participants, clinicians, and the technology in context, takes time and effort. It takes ongoing work from the study staff to help the technology align with the needs of users, who are part of different populations, in a particular context. The daily work of healthcare staff in troubleshooting the technology can become invisible, instead of helping to inform future implementation and service delivery [19]. This is important to consider when thinking about how staff are trained to work with digital health interventions. In the WHO guidelines [1], training time for healthcare staff is presented as a finite number of hours. Yet, due to the changeable nature of digital health technology and the associated practices that come with it, our findings show that presenting this number as finite, and occurring within a certain span of time, could be misleading. Based on our insights and more reflective of the effort, strategies and time needed to make a digital health intervention work, we propose different questions and aspects to assess what it takes to have staff integrate digital technology into daily care. For example:

- How do staff deal with challenges on a day-to-day basis, who do study staff ask for help, and what kinds of tinkering efforts are involved in making the technology work?
- How do patients/clinicians/study staff share information or strategies with one another for making the technology work and what (if any) additional resources do they require aside from training?
- How is the expertise of the clinicians aligned with, enhanced or eroded by the technology and vice versa (i.e., how, if at all, do the technology and clinicians work synergistically)?
- What visions of use exist for the digital health intervention across various users, how do these visions of use change over time and in practice, and what does this mean for how people work collaboratively (or not) with the digital health technology?

In suggesting these evaluation questions, we do not mean to say that the metrics mentioned in WHO guidelines, or that certain methodological approaches, have no value. They can surely provide insight into challenges or successes related to aspects of digital health interventions. However, as Greenhalgh and Russell [8] put it when discussing eHealth evaluation, “the tricky questions are more philosophical and political than methodological and procedural” (p4). A 2020 WHO guide entitled “Digital implementation investment guide (DIIG): integrating digital interventions into health programmes” [20], acknowledges the importance of multiple users and their various needs, as well as the time intensive nature of training and implementation of digital interventions within broader health systems. However, the monitoring and evaluation aims tend to fall into a technically focused evaluation that obscures the changeable nature of the intervention in which collaborative work between the technology, its multiple users, and the changing environment co-produce an intervention that works. It enlists a model of technological maturity, which focuses on the feasibility, usability, efficacy etc. of the digital technology as a rather linear process where the technology either works and goes on, or fails and must be rethought or thrown out. If we assess a digital health intervention based on these metrics and conclude that the intervention is working, usable, efficacious, or effective in relation to a “static” or final app, user, and technical system, we do not actually say much about whether the intervention will be successful in a particular context. That is because the elements change and are co-produced throughout the process of evaluation and monitoring by the people (e.g., research participants, healthcare workers, app developers, researchers), places (e.g., home, clinic, Cape Town, Montreal) and things (e.g., phones, tablets, self-tests, regulations) working with and around the intervention. Similar to the insights of Marent and colleagues [21], we show that people are not consistently accepting or rejecting digital health technology, as people’s envisioned and actual use of the technology change over time.

Finally, we would argue that developing and implementing a digital health intervention, such as an app-based digital program, which is easy to use, communicates exactly as intended, and works on its own to produce health outcomes, is not necessarily the goal when we conceptualize digital health as an actor contributing to good care during HIV testing. HIVST and subsequent care is not a process that is necessarily easy or straightforward for a person who tests. It can be stressful, difficult, or scary. It takes time. Care is collaborative and involves interactions between multiple, shifting, people and technologies [22–24]. The ways in which we evaluate digital health technology, and how these evaluations inform implementation of these technologies, should reflect the goal of providing good care and not the success of the digital health technology on its own. What constitutes good care during diagnosis with digital health technology is continuously reshaped based on the needs of the changing patient, healthcare provider, technology, and environment. Instead, we should aim to explore the multiple, changing visions of digital health technology use, while also accounting for how other actors in the environment change and work collaboratively with the digital health technology to make good care in practice. We hope that this paper provides insights for those planning future implementation and evaluation of related technology within an ever-changing landscape of stakeholders, devices, technical infrastructure, and health policy. Failing to recognize the evolving nature of digital health technology and tech-based programs and the shifting roles and interactions between relevant actors and end-users during implementation of these technologies is akin to ignoring key components of a theory of change and could risk future success of digital health technology programs in any setting.

## Supporting information

S1 Checklist Inclusivity in global research. (DOCX)
